# Unravelling drug resistance in leishmaniasis: genomic adaptations and emerging therapies

**DOI:** 10.3389/fmolb.2025.1573618

**Published:** 2025-05-26

**Authors:** Chandra Kanta Bhusal, Shweta Sinha, Davinder Kaur, Rakesh Sehgal

**Affiliations:** ^1^ Department of Medical Parasitology, Post Graduate Institute of Medical Education and Research, Chandigarh, India; ^2^ Department of Pharmacology and Therapeutics, All India Institute of Medical Sciences, Gorakhpur, India; ^3^ Department of Microbiology, Aarupadai Veedu Medical College and Hospital, Puducherry, India

**Keywords:** leishmaniasis, drug resistance, genomic plasticity, antileishmanial therapy, drug targets

## Abstract

Leishmaniasis remains a significant global health challenge, with over a billion people at risk of infection and limited effective treatment options due to escalating drug resistance. This review explores the underlying mechanisms of drug resistance in *Leishmania* species, focusing on genomic plasticity as a driving factor for survival and adaptation. Key mechanisms, including genetic mutations, gene amplification, chromosomal rearrangements, and efflux transporters, contribute to the parasite’s ability to evade existing therapies. Advances in genomic and proteomic studies have provided deeper insights into these resistance pathways, enabling the development of novel therapeutic strategies. Additionally, this review highlights current therapeutic approaches, including combination therapies and potential new drug candidates, that address multidrug resistance and explore the vulnerabilities of *Leishmania*. Understanding these mechanisms and their clinical implications is essential for developing targeted interventions that improve treatment outcomes and combat resistance in leishmaniasis.

## 1 Introduction

Leishmaniasis is a parasitic disease caused by protozoa of the genus *Leishmania* and transmitted through the bites of infected female phlebotomine sandflies. More than 90 sandfly species are known to transmit *Leishmania* parasites. The disease is widespread, affecting approximately89 countries (Torres-Guerrero et al., 2017). It is endemic in Asia, Africa, the Americas, and the Mediterranean region, placing over 1 billion people at risk of infection (World Health Organization, 2025). Leishmaniasis presents in four clinical forms: cutaneous, mucocutaneous, visceral (kala-azar), and post-kala-azar dermal leishmaniasis (Torres-Guerrero et al., 2017). However, many infections remain asymptomatic (Singh et al., 2020). *Leishmania* can also act as an opportunistic pathogen in immunosuppressed individuals.

Chemotherapy remains the cornerstone of leishmaniasis management and control. Treatment choice depends on multiple factors, including disease type, coexisting conditions, parasite species, and geographic location. Since current drugs cannot fully eliminate the parasite, immunocompetence is crucial to prevent relapse. Pentavalent antimonials, amphotericin B, paromomycin, miltefosine, pentamidine, and sitamaquine are the medications used in current treatments regimen (Kapil et al., 2018). Each drugs have its own mechanism of action, such as antimonials are believed to interfere with the parasite’s energy production, inhibiting glycolysis and fatty acid oxidation. They may also disrupt parasite thiol metabolism, leading to oxidative stress and cell death (Haldar et al., 2011), Amphotericin B binds to ergosterol, forming pores in the membrane and causing parasite death (Stone et al., 2016). Miltefosine disrupts cell membrane integrity and inhibits phospholipid metabolism. It also interferes with mitochondrial function and triggers apoptosis-like cell death in the parasite (Palić et al., 2019). In many areas, antimonials continue to be the major medication used to treat various types of leishmaniasis. However, antimony resistance has made the use of substitute drugs necessary, particularly in the Indian subcontinent. Currently, parenteral paromomycin, amphotericin B (AmB), and the oral miltefosine (MIL) are widely used. The frequency of treatment failure may be significant in patients treated with MIL, which has supplanted antimonials in the kala-azar extermination campaign in nations such as India, even though it has been noted in patients treated with the majority of anti-leishmanials. AmB is highly efficacious but also has associated toxicity such as includes fever, nausea, vomiting, rigors, hypertension or hypotension, and hypoxia when administered in its free deoxycholate form which has been overcome in its liposomal formulation (Laniado-Laborín and Cabrales-Vargas, 2009; Zhang et al., 2025). Unfortunately, resistance to AmB has also been shown to be a concern in laboratory experiments.

**FIGURE 1:**
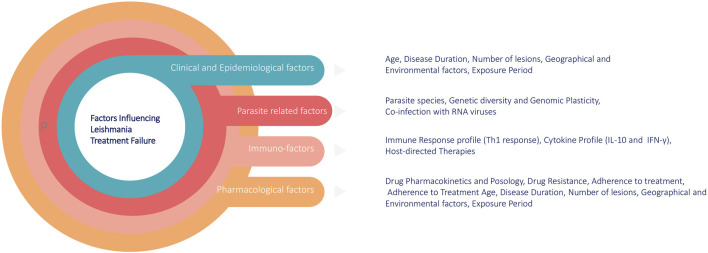
Factors contributing to treatment failure and drug resistance in leishmaniasis.

The rise of drug resistance impacts treatment outcomes and is influenced by multiple factors. These include host-related factors (e.g., immune response and cytokine profile), immuno-factors, drug pharmacokinetics (e.g., metabolism and adherence to treatment), and parasite-specific factors, such as genetic plasticity and co-infections (Figure 1). A comprehensive understanding of these factors is crucial for developing targeted interventions to overcome treatment failure. Moreover, the numbers of leishmaniasis cases are increasing worldwide. Some reasons are the lack of vaccines, difficulties in controlling vectors and the increasing number of parasites resistance to chemotherapy. The rise of drug resistance impacts treatment outcome, and understanding its causes, spread, and impact will help us manage the risks it imposes (Moncada-Diaz et al., 2024). Out of various reasons of appearance of drug resistance, genome plasticity is another key factor in the survival of Leishmania parasites and their development of drug resistance in which genetic variations such as mutations, gene amplifications, and chromosomal rearrangements play crucial roles in the appearance of resistance (Kamran et al., 2023). In the following sections we discuss about the *Leishmania* treatment regimens, mechanism behinds underlying drug resistance to currently available antileishmanial drugs, role of genetic adaptability in drug resistance and currents drugs that are under drug discovery pipelines and potential drug targets.

## 2 Leishmaniasis treatment regimens

Pentavalent antimonials (SbV) have been the cornerstone in the treatment of leishmaniasis for many decades. The two primary forms used are sodium stibogluconate (SSG) and meglumine antimoniate (MA), each administered as parenteral drugs (IM, IV, or IL) at a standard dose of 20 mg/kg/day for 28–30 days (Haldar et al., 2011). In the 1980s, the World Health Organization (WHO) recommended the use of SbV at an increased dose of 20 mg/kg/day up to a maximum of 850 mg for 20–30 days. Despite their long-standing use and initial success, their effectiveness has gradually declined due to widespread resistance and adverse effects. The main drawback of pentavalent antimonials is their severe toxicity, including cardiotoxicity (e.g., ventricular tachycardia, prolonged QTc interval), pancreatitis, pancytopenia, and nephrotoxicity. Due to these risks, SbV is not recommended for HIV-VL co-infected patients as they experience higher toxicity and reduced efficacy (Haldar et al., 2011). Due to the increasing resistance and toxicity of SbV, alternative treatments such as paromomycin (PM) have gained prominence in managing leishmaniasis. Paromomycin (PM), an aminoglycoside antibiotic, is an affordable and effective option for treating leishmaniasis, administered intramuscularly at 15 mg/kg/day for 21 days. Although PM shows high cure rates (94.6% in a Phase III study in India), its prolonged treatment duration poses a challenge in endemic areas. PM is also potentially nephrotoxic and ototoxic. While it is used alone for visceral leishmaniasis (VL) in some regions, in Africa, it is typically combined with SSG. Topical formulations of PM are used for cutaneous leishmaniasis (CL), though efficacy varies by geographical region, with better outcomes for systemic use in Brazil (Pokharel et al., 2021). While PM remains a viable option, particularly in combination therapies, another potent alternative is Amphotericin B, which is particularly effective in cases resistant to pentavalent antimonials.

Amphotericin B, a polyene antifungal, is highly effective for treating leishmaniasis, particularly in regions with pentavalent antimonial resistance. Administered intravenously at 0.75–1 mg/kg/day for 15–20 days, it has cure rates approaching 100%. However, its use is limited by severe nephrotoxicity and the need for hospitalization during treatment. Liposomal formulations like L-AmB (AmBisome) offer targeted delivery with fewer side effects, making it the preferred treatment for visceral leishmaniasis, especially in HIV-VL co-infections. Despite its high cost and need for a cold chain, L-AmB’s safety profile and variable dosing regimens make it a versatile option across different geographical regions (Frézard et al., 2022). *Given the challenges associated with injectable therapies like Amphotericin B, the development of oral treatments such as Miltefosine (MIL) has significantly impacted leishmaniasis management.* Miltefosine (MIL) is the first effective oral drug for visceral leishmaniasis, with a 94% cure rate. Initially introduced in 2002 and widely used in India’s kala-azar elimination program, it is now registered in several countries. Administered at 2–2.5 mg/kg for 28 days, MIL works by increasing nitric oxide production in macrophages, disrupting parasite membranes, and damaging mitochondria. However, its long half-life and poor compliance contribute to resistance development. MIL is teratogenic and unsuitable for pregnant women, with common side effects including gastrointestinal issues, renal toxicity, and dehydration (Scarpini et al., 2022) (Table 1).

## 3 Geographic variations in antileishmanial drug resistance

Treatment options for leishmaniasis vary significantly by region due to differences in dominant *Leishmania* species and emerging drug resistance patterns (Ponte-Sucre et al., 2017). In South Asia, *Leishmania donovani* shows high resistance to sodium stibogluconate (SSG) and declining susceptibility to miltefosine, necessitating alternatives like liposomal amphotericin B (L-AmB) and combination therapies. In East Africa, SSG remains a primary treatment, though resistance has been reported, prompting the use of SSG-paromomycin combinations. In Latin America, where *Leishmania braziliensis* and related species prevail, antimonials like meglumine antimoniate are still used, but resistance varies by region, with pentamidine and miltefosine serving as alternatives. The Mediterranean and Middle East face challenges with *Leishmania infantum* and *Leishmania tropica*, where antimonial resistance is emerging, leading to increased reliance on L-AmB. Central Asia reports growing SSG resistance in *L. tropica*, pushing the adoption of thermotherapy and L-AmB (Pigott et al., 2014; Herrera et al., 2020) (Table 2).

**TABLE 1:** Drug combinations and efficacy in the treatment of leishmaniasis.

Drug combination	Dose	Duration (Days)	*Leishmania* species	Efficacy results	References
SSG + PM	SSG: 20 mg/kg daily	17	*L. donovani*	Higher cure and survival rates compared to SSG alone	Melaku et al. (2007)
MIL + AmBisome	AmBisome: 5 mg/kg single dose; MIL: 2.5 mg/kg/day	7	*L. donovani*	Cure rate >95%, compared to 91% with AmBisome alone	Sundar et al. (2008)
PM + AmBisome	AmBisome: 5 mg/kg single dose; PM: 15 mg/kg/day	10	*L. donovani*	Cure rate >97%	Sundar et al. (2011a)
AmBisome + SSG	AmBisome: 10 mg/kg single dose; SSG: 20 mg/kg/day	10	*L. donovani*	Definitive cure rate of 87%	Wasunna et al. (2016)
AmBisome + MIL	AmBisome: 10 mg/kg single dose; MIL: 2.5 mg/kg/day	10	*L. donovani*	Definitive cure rate of 77%	Wasunna et al. (2016)
SSG + PM	SSG: 20 mg/kg daily	60+	*L. aethiopia*	Effective for diffuse CL.	Sundar et al. (2019)
SSG + Allopurinol	SSG: 20 mg/kg daily	15	*L. tropica*	Recommended for treating leishmaniasis recidivans	Sundar et al. (2019)
SSG + Pentoxifylline	SSG: 20 mg/kg daily	10–20	*L. major*	Effective for treating *L. major*	Sundar et al. (2019)
SSG + Pentoxifylline	SSG: Dose not specified; Pentoxifylline: 400 mg t.i.d	10–20	*L. braziliensis*	Not more effective than SSG monotherapy	Brito et al. (2017)
SSG + Pentoxifylline	SSG: Dose not specified	10–20	MCL (Mucocutaneous Leishmaniasis)	High cure rates observed in small studies	Lessa et al. (2001), Machado et al. (2007)
PMM + MIL	PMM: 350 mg/kg/day, MIL: 20 mg/kg/day	2	*L. infantum*	Not more than effective than PMM alone	Hendrickx et al. (2017)
Tamoxifen + Amphotericin B	Tamoxifen: 6.5 mg/kg/day, Amphotericin B: 1.2 mg/kg/day	Not specified	*L. amazonensis*	Significant reduction in lesion size and parasite burden compared to monotherapy and untreated group	Trinconi et al. (2014)
PM + CQ	PM: 50 mg/kg/day, CQ: 25 mg/kg/day	10	*L. major, L. mexicana*	Slight reduction in lesion size for both species, but no significant additional reduction in parasite load compared to PM alone	Wijnant et al. (2017)
SbV + Topical/Oral Tamoxifen	Oral: Tamoxifen 40 mg/day; Topical: 0.1% tamoxifen citrate; SbV: 20 mg SbV/kg/day	20	*L. braziliensis*	Cure rates: Oral 58%, Topical 36.4% after 6 months, well tolerated. Higher cure rates with oral tamoxifen in comparison with SbV alone (40%)	Machado et al. (2018)
AmBisome + Miltefosine/AmBisome + Paromomycin/Paromomycin + Miltefosine/AmBisome (monotherapy)	AmBisome: 5 mg/kg single dose + Miltefosine: 2.5 mg/kg/day (7 days)/AmBisome: 5 mg/kg single dose + Paromomycin: 15 mg/kg/day (10 days)/Paromomycin: 15 mg/kg/day + Miltefosine: 2.5 mg/kg/day (10 days)/AmBisome: 5 mg/kg on days 1, 3, and 5	5–10 days depending on combination	Visceral Leishmaniasis	Cure rates were: AmBisome + Miltefosine: 94.4%, AmBisome + Paromomycin: 99.4%, Paromomycin + Miltefosine: 97.9%, and AmBisome monotherapy: 98.1%. Minor adverse events were observed across groups, but no relapses or post-kala-azar dermal leishmaniasis (PKDL) occurred within the 6-month follow-up. The combinations provided similar or superior outcomes compared to AmBisome monotherapy	Rahman et al. (2017)
Paromomycin + Miltefosine (PM/MF)/Sodium Stibogluconate + Paromomycin (SSG/PM)	PM: 20 mg/kg + MF: allometric dose/SSG: 20 mg/kg + PM: 15 mg/kg/day	14 days (PM/MF), 17 days (SSG/PM)	Visceral Leishmaniasis (VL)	In this comparative study, definitive cure rates at 6 months were 91.2% for PM/MF and 91.8% for SSG/PM, narrowly missing the noninferiority margin (7%) in mITT analysis but demonstrating noninferiority in the per-protocol analysis. Both treatments were well tolerated, with only 4 serious adverse events related to the study drug, including 1 SSG-related death. PM/MF was more patient-friendly, with fewer injections, shorter treatment duration, and no risk of life-threatening cardiotoxicity	Musa et al. (2023)
Liposomal Amphotericin B+ Miltefosine/Miltefosine Monotherapy	Liposomal Amphotericin B: 7.5 mg/kg (single dose) + Miltefosine: 2.5 mg/kg/day/Miltefosine: 2.5 mg/kg/day	14 days (combination), 28 days (monotherapy)	Visceral Leishmaniasis (VL)	In this randomized trial, clinical and parasitological cure rates at the end of therapy were 100% for both groups. At 6 months follow-up, 17.4% of patients in the miltefosine monotherapy group experienced relapse, while none in the combination group did. Over 5 years, 10 patients in the miltefosine group developed post–kala-azar dermal leishmaniasis (PKDL), while none in the combination therapy group experienced this	Goswami et al. (2020)

**TABLE 2:** Regional epidemiology and treatment approaches for leishmaniasis.

Region	Predominant species	Clinical forms	Epidemiological features	First-line treatment	Alternative treatments	Resistance patterns	References
Mediterranean Basin	*L. infantum*	VL, CL	Zoonotic; dogs as main reservoir; *Phlebotomus* spp. vectors	Liposomal Amphotericin B	Meglumine antimoniate, Miltefosine	Low-level resistance to antimonials	World Health Organisation (2023), Scarpini et al. (2022)
East Africa	*L. donovani*	VL (kala-azar)	Anthroponotic; post-kala-azar dermal leishmaniasis (PKDL) common	Sodium stibogluconate (SSG) + Paromomycin combination	Liposomal Amphotericin B, Miltefosine	Increasing SSG resistance (30%–60% in some areas)	Jones and Welburn (2021)
South Asia (India, Nepal, Bangladesh)	*L. donovani*	VL	Anthroponotic; high population density; indoor transmission	Liposomal Amphotericin B, Miltefosine	Paromomycin, Amphotericin B deoxycholate	High antimonial resistance (>60%); emerging miltefosine resistance	Rijal et al. (2019)
Latin America	*L. infantum* (VL), *L. braziliensis*, *L. mexicana*, *L. amazonensis*	VL, CL, MCL	Zoonotic; sylvatic and peridomestic cycles	Meglumine antimoniate	Amphotericin B formulations, Pentamidine	Variable antimonial resistance	Ponte-Sucre et al. (2017)
Middle East/Central Asia	*L. major*, *L. tropica*	CL	Zoonotic (*L. major*) and anthroponotic (*L. tropica*)	Meglumine antimoniate, Sodium stibogluconate	Liposomal Amphotericin B, Local therapies	Moderate antimonial resistance	Yurchenko et al. (2023)
Southern Europe	*L. infantum*	VL, CL	Zoonotic; increasing cases in immunocompromised patients	Liposomal Amphotericin B	Miltefosine	Low resistance levels	Tunalı and Özbilgin (2023)

## 4 Molecular mechanisms of drug resistance in *Leishmania*

### 4.1 Mechanism underlying antimony resistance in *Leishmania*

One of the primary mechanisms of antimony resistance in *Leishmania* is the reduced uptake of the drug by the parasite. Aquaglyceroporin 1 (AQP1) is known to facilitate the uptake of SbIII by the parasite. In drug-resistant parasites, downregulation of AQP1 has been observed, leading to decreased drug uptake and subsequent resistance (de Santana et al., 2025; Hefnawy et al., 2017; Santos et al., 2023). Another significant mechanism involves increased intracellular thiol levels. In drug-sensitive strains, SbIII disrupts thiol homeostasis by inducing the efflux of thiols such as trypanothione (TSH), glutathione (GSH), and cysteine, which maintain thiol redox homeostasis in *Leishmania*, protecting the parasite from chemical and oxidative stress. The γ-GCS gene encodes an enzyme catalyzing the rate-limiting step of GSH biosynthesis, while the ODC gene encodes an enzyme regulating polyamine biosynthesis. Polyamines are precursor metabolites of trypanothione. Antimony-resistant strains have shown inconsistent upregulation of γ-GCS and overexpression of ODC genes, increasing the intracellular thiol-dependent antioxidant capacity and resulting in resistance to antimony. Additionally, the trypanothione reductase gene is amplified in antimony-resistant isolates, leading to high intracellular trypanothione levels and increased resistance to SbIII (Haldar et al., 2011; Fekrisoofiabadi et al., 2019).

Sequestration and rapid drug efflux also contribute to antimony resistance. ATP-binding cassette (ABC) transporters efflux the drug out of the parasite or sequester it in intracellular vesicles. The two classes of ABC transporters involved in this process are P-glycoprotein (e.g., MRPA) and multi-drug resistance-related protein (e.g., MRP1). Genes encoding these transporters are amplified in antimony-resistant parasites, leading to effective drug efflux and sequestration (Berg et al., 2015). Changes in membrane fluidity have been demonstrated in resistance to antimony combinations, further contributing to the resistance mechanism (Salari et al., 2022). Modulation of cell death through heat shock proteins (e.g., HSP83 and HSP70) has been reported in resistant parasites. Additionally, cell death-related proteins such as protein tyrosine phosphatase (PTP), proliferating cell nuclear antigen (PCNA), and mitogen-activated protein kinase (MAPK) show differential expression, with PTP and PCNA being upregulated and MAPK being downregulated in antimony-resistant strains (Salari et al., 2022; Tandon et al., 2014).

Beyond intrinsic parasite resistance mechanisms, *Leishmania* also modulates signaling pathways in host macrophages. Drug-resistant parasites have been shown to alter the host-pathogen interaction and the host immune response, contributing to the development of resistance (Mukhopadhyay et al., 2011). Certain proteins are differentially expressed in response to antimony. For example, proteins such as histone 1, H2A, H4, and leucine-rich repeat protein are overexpressed in antimony-resistant parasites, while proteins like the kinetoplastid membrane protein (KMP-11) are under-expressed (Das et al., 2015). Apart from genetic and biochemical adaptations, external factors such as the misuse of antimony drugs has significantly contributed to the development of resistance. Practices such as inadequate dosing, inappropriate treatment regimens, the free availability of drugs, management of patients by unqualified persons, and incomplete treatment courses have led to the development of subtherapeutic levels of antimony in the blood, promoting parasite tolerance and resistance to the drug (Kazemi-Rad et al., 2013).

Further reinforcing these resistance mechanisms, a study on *L. donovani* compared Sb(V)-sensitive and -resistant strains from kala-azar patients. The resistant strain exhibited cross-resistance to miltefosine and other drugs. Proteomic analysis identified altered programmed cell death (PCD) pathways as central to resistance. Notably, the heat shock protein HSP83 was found to enhance drug resistance by disrupting mitochondrial membrane potential and diminishing drug-induced PCD. Conversely, the protein SKCRP14.1 promoted PCD in response to antimonials but conferred protection against miltefosine-induced PCD (Ennes-Vidal et al., 2017).

### 4.2 Complex mechanisms of miltefosine resistance in *Leishmania*

Recent studies have identified multiple mechanisms underlying miltefosine (MF) resistance in *Leishmania* species, posing significant challenges to effective treatment. This resistance involves genetic, biochemical, and immunological factors that reduce the drug’s efficacy. A study conducted by Caroline R. Espada et al. (2019) on *Leishmania (V.) braziliensis* clinical isolates found that decreased MF susceptibility was linked to reduced drug accumulation. This reduction was attributed to diminished Ros3 mRNA expression rather than polymorphisms in MT-Ros3 complex genes, which are crucial for drug uptake. This finding underscores the need for new molecules or modifications to MF to overcome MT-Ros3 dependence and enhance treatment efficacy (Espada et al., 2019). Beyond reduced drug accumulation, MF resistance in *Leishmania* exhibits remarkable stability, as indicated by a consistent EC50 value, even after prolonged culture without drug exposure. This suggests a specific and persistent mechanism likely involving changes in transporter expression or translocation machinery. Notably, this resistance is largely exclusive to MF, with some exceptions showing reduced susceptibility to SbIII, hinting at a complex interplay between different resistance pathways (Vacchina et al., 2016).

The overexpression of multidrug resistance proteins, such as MRPA, is another critical factor in MF resistance. MRPA, an ATP-binding cassette (ABC) transporter, actively pumps MF out of the parasite’s cells, thereby reducing its intracellular concentration and effectiveness (Khanra et al., 2017). Additionally, other ABC transporters like ABCB4, ABCG4, and ABCG6 contribute to this resistance by enhancing drug efflux (Pérez-Victoria et al., 2011; Da Costa et al., 2018). MF resistance is also influenced by immune evasion strategies, as seen in *L. donovani*. This impairment weakens the host’s ability to mount an effective immune response, characterized by a lack of Th1-type immune responses and reduced production of essential cytokines and antibodies. Consequently, the host struggles to control and eliminate the parasite, even with MF treatment, complicating treatment outcomes and resistance management (Khanra et al., 2017).

In *Leishmania infantum*, resistance has been linked to the deletion of the MSL locus, affecting key enzymes (NUC1 and NUC2) involved in MF susceptibility. This deletion also leads to increased baseline lipid content, including ergosterol, which may act as a reservoir for MF, contributing to resistance. Moreover, isolates from relapsed patients demonstrated better control of lipid perturbations and nitric oxide accumulation in macrophages, suggesting a role in modulating host immune responses (Carnielli et al., 2022). Expanding on these species-specific variations, whole genome sequencing of *L. donovani* identified a significant mutation in the LdMT gene, leading to transporter inactivation. This mutation, along with changes in membrane fluidity and gene expression—such as the upregulation of genes related to surface proteins and phosphoglycan biosynthesis, and the downregulation of stress response and folate transport genes—highlights specific genetic and metabolic adaptations rather than a general resistance profile. Despite reduced metacyclogenesis, these resistant parasites maintain their ability to invade and replicate in host cells (Vacchina et al., 2016). Together, these findings underscore the multifaceted nature of MF resistance in *Leishmania*, integrating genetic mutations, metabolic alterations, and immune evasion strategies, significantly impacting treatment efficacy. Understanding these mechanisms is crucial for addressing the growing challenge of drug resistance in leishmaniasis.

### 4.3 Sterol mutations and amphotericin B resistance in *Leishmania*

Resistance to amphotericin B (AmB) in *Leishmania* primarily involves mutations in the sterol biosynthesis pathway, resulting in altered membrane sterol composition. Key mutations in genes such as C24SMT, C5DS, and C14DM lead to the loss of ergosterol and the accumulation of other sterol precursors, which reduce the binding affinity of AmB to the parasite membrane and decrease drug sensitivity (Mukherjee et al., 2020). This resistance mechanism has been observed in both laboratory-generated resistant strains and clinical isolates, underscoring its relevance in both experimental and field settings (Pountain et al., 2019).The altered sterol profile, often featuring ergosta-7,22-dienol or cholesta-5,7,22-trienol, compromises the drug’s efficacy, indicating that mutations in sterol metabolism are central to AmB resistance in *Leishmania* (Yao and Wilson, 2016; Alpizar-Sosa et al., 2022). Further studies have shown that resistance is associated with an increased conversion of β-sitosterol into stigmasterol, significantly raising the IC50 by four times compared to wild-type strains. This sterol alteration, observed in both promastigotes and axenic amastigotes, highlights stigmasterol’s role in AmB resistance, despite the reduced infectivity of the resistant strain *in vitro* (Bansal et al., 2020).

**FIGURE 2:**
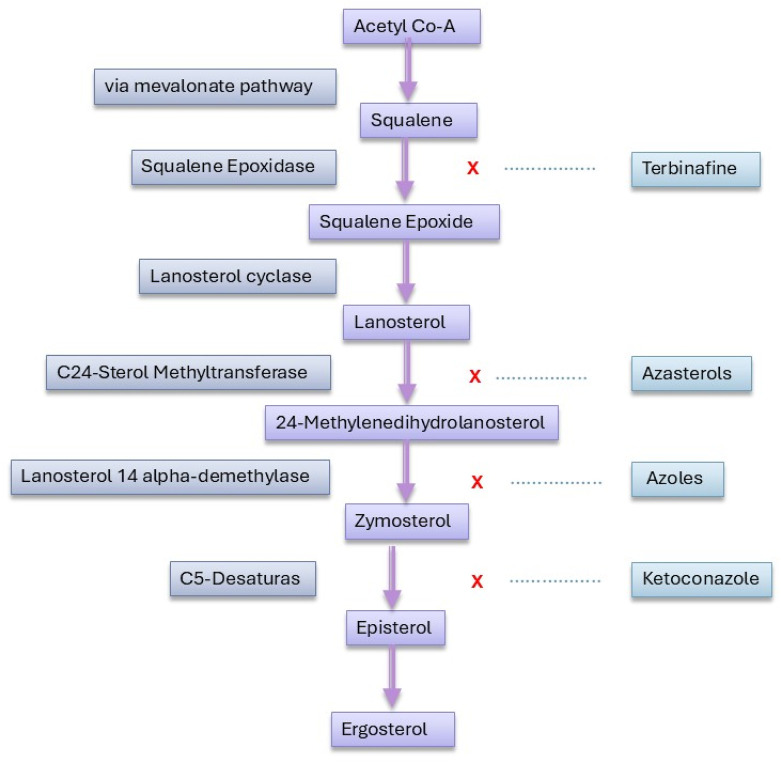
Enzymatic drug targets and inhibition sites in the sterol biosynthesis pathway of *Leishmania*.

Additionally, in *L. martiniquensis*, AmB resistance is linked to increased metacyclogenesis, growth, and infectivity, with resistant strains persisting longer in mice without causing clinical disease. These asymptomatic hosts could act as reservoirs, enhancing transmission, which underscores the need for vigilant monitoring of AmB-resistant *Leishmania martiniquensis*, particularly in relapsing and HIV-coinfected patients (Mano et al., 2023). The sterol biosynthesis pathway depicted in Figure 2 highlights how alterations at key enzymatic steps can lead to the production of alternative sterols that diminish AmB efficacy. Moreover, other studies have found that *Leishmania* can develop resistance to AmB through the loss of ergosterol and its replacement with cholestane-type sterols. This process, involving mutations in the enzyme sterol 14α-demethylase, disrupts sterol synthesis and affects AmB binding. Additionally, these resistant strains exhibit increased sensitivity to oxidative stress (Mwenechanya et al., 2017). Other studies have identified mutations in sterol biosynthesis enzymes, such as SMT, as significant contributors to AmB resistance. These mutations can alter sterol composition and membrane properties, affecting AmB binding and efficacy. Additionally, resistance mechanisms have been linked to changes in the miltefosine transporter and increased membrane fluidity (Pountain et al., 2019).

Further research suggests that resistance mechanisms to AmB in *Leishmania* involve complex interactions between genetic mutations and metabolic changes. For instance, AmB-resistant *Leishmania* lines show decreased levels of oligohexoses, which may influence virulence, and increased levels of protective thiols such as trypanothione and glutathione. These findings indicate that metabolic adaptations, beyond primary genetic mutations, play a crucial role in shaping the resistance profile of *Leishmania* parasites (Pountain and Barrett, 2019). Additionally, it has been found that the enzyme L-asparaginase (LdAI) is crucial for *L. donovani*’s resistance to AmB, with its overexpression enhancing survival under treatment (Singh et al., 2017). Elevated levels of the protein Sir2 in resistant parasites lead to increased MDR1 expression, enhanced drug efflux, reduced ROS levels, and decreased apoptosis, contributing to higher resistance to AmB. Conversely, inhibiting or deleting Sir2 increases drug susceptibility, making Sir2 a potential resistance marker for visceral leishmaniasis (Purkait et al., 2015). Lastly, some studies suggest that *Leishmania* resists AmB by protecting against membrane ion leakage and oxidative damage, despite normal ergosterol levels. This resistance mechanism involves altered cell signalling due to AmB’s membrane-thinning effects (Cohen, 2016).

### 4.4 Physiological and genetic adaptations to paromomycin resistance in *Leishmania*

**FIGURE 3:**
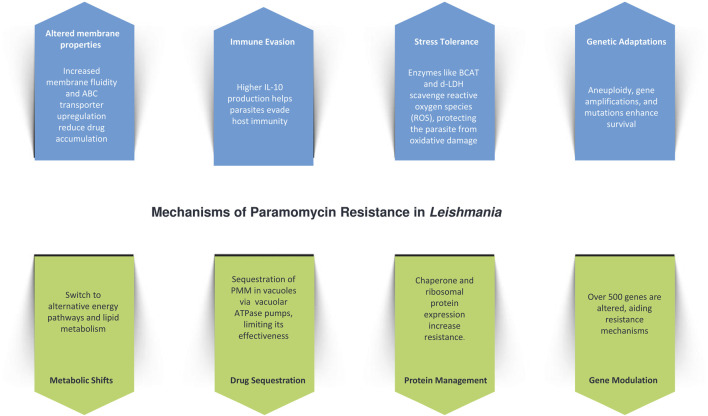
Mechanistic insights into Paromomycin resistance in *Leishmania*.

Paromomycin (PMM) resistance in *Leishmania* is driven by a combination of physiological, genetic, and metabolic adaptations (Figure 3). One of the key mechanisms is increased membrane fluidity, which impairs drug penetration and decreases intracellular accumulation, thereby reducing the drug’s effectiveness. This is accompanied by the upregulation of ATP-binding cassette (ABC) transporters, such as MDR1 and MRPA, which enhance drug efflux and further contribute to resistance. The resistant parasites also exhibit improved tolerance to host defense mechanisms, such as nitrosative stress, and evade immune responses through increased interleukin-10 (IL-10) production, which facilitates immune evasion (Shaw et al., 2019).

In addition to drug efflux and membrane adaptations*, L. donovani* reinforces its resistance by withstanding nitric oxide (NO) stress, particularly in the amastigote stage, a critical phase for survival within host macrophages (Hendrickx et al., 2014). This is supported by metabolic shifts, including the upregulation of enzymes like branched-chain aminotransferase (BCAT) and d-lactate dehydrogenase (d-LDH), which help scavenge reactive oxygen species (ROS) and protect the parasite from oxidative damage (Rastrojo et al., 2018). Genetic adaptations, such as aneuploidy and ribosomal RNA gene amplifications, enhance these defense mechanisms. Whole-genome sequencing of resistant clones has revealed no single mutation consistently linked to resistance, but multiple genetic variations—such as single nucleotide variants (SNVs) and copy number variations (CNVs)—have been observed. These variations are concentrated in genes related to protein synthesis, mitochondrial function, and virulence factors like HSP78 and sterol 24-C-methyltransferase, along with CNVs affecting mitochondrial transport, vesicular trafficking, and protein turnover (Shaw et al., 2019; Hendrickx et al., 2021).

Metabolic adaptations are also central to PMM resistance, with resistant parasites exhibiting a shift away from oxidative phosphorylation towards glycosomal succinate fermentation, and increased reliance on lipid and amino acid metabolism for energy. Reduced DNA synthesis paired with enhanced DNA repair, alongside decreased protein synthesis and degradation, further support survival under drug pressure. Transcriptomic analysis has shown modulation of over 500 genes, while calcium channel antagonists, such as verapamil and amlodipine, increase PMM susceptibility, implicating ABC transporters in the resistance pathway. Interestingly, while PMM-resistant parasites modulate NO levels in infected macrophages, ROS levels remain unaffected (Verma et al., 2017).

Another significant aspect of PMM resistance involves drug sequestration. PMM is internalized via endocytosis and sequestered in vacuoles, where vacuolar ATPase pumps are upregulated to isolate the drug, reducing its efficacy. The stress induced by PMM leads to the increased expression of chaperone proteins, which aid in protein folding and turnover, helping the parasite cope with the drug’s effects. Additionally, the upregulation of ribosomal proteins enhances protein synthesis, and glycolytic enzyme overexpression boosts energy production, collectively contributing to the parasite’s survival and resistance (Chawla et al., 2011).

A comparative overview of resistance mechanisms across major antileishmanial drugs is provided in Table 3, highlighting recurrent themes such as transporter upregulation, metabolic adaptations, and genetic mutations.

**TABLE 3:** Studies on recent research on *Leishmania* drug resistance.

Drug	Treatment	Strain	Resistance strain	Main findings	Resistance mechanism	References
Amphotericin B	Liposomal formulation, single dose	*L. mexicana*	Amphotericin B-resistant	Ergosterol diminished in resistant strains; alternative sterols present	N176I mutation in sterol 14α-demethylase (CYP51)	Mwenechanya et al. (2017)
Amphotericin B	—	*L. mexicana*, *L. infantum*	AmBR (resistant to AmB), NysR (resistant to Nystatin)	Fourteen L. mexicana and one L. infantum line developed AmB or nystatin resistance, with sterol modifications and oxidative stress induction in rich medium	Mutations in C24SMT, C5DS, and deletion of miltefosine transporter	Alpizar-Sosa et al. (2022)
Miltefosine	Visceral Leishmaniasis (VL)	*L. donovani* (Indian subcontinent)	Miltefosine-resistant *L. donovani*	Genomic microarray analysis identified 311 differentially expressed genes (∼3.9% of the genome). These genes are involved in metabolic pathways, transporters, and cellular components	Mechanisms include compromised DNA replication/repair, reduced protein synthesis and degradation, altered energy utilization, increased drug efflux, and enhanced antioxidant defense (via trypanothione metabolism)	Kulshrestha et al. (2014)
Antimony	SbV, SbIII	*L. donovani*	Various clinical isolates (9515, 9518, 9551)	AQP1 indels and MRPA CNVs contribute to antimony resistance; other mechanisms may also be involved	Gene deletion, SNPs, TC indel in AQP1 disrupting reading frame, MRPA CNV amplification, SNVs, CNVs	Potvin et al. (2021)
Antimonials (Sb)	Stepwise drug-resistance selection and targeted gene disruption	*L. infantum*	*mrpA−/-* mutant strain and wild-type (*L. infantum*)	Five independent *mrpA−/-* mutants were selected for Sb resistance, showing changes in ploidy and amplifications of chromosome 23. SNP analyses revealed mutations in SAT gene (*satQ390K*, *satG321R*, *satG325R*), leading to increased Sb resistance	Overexpression of ABCC2, not ABCC1, resulted in increased Sb tolerance in *mrpA−/-* mutants. SAT mutations (*satG321R*, *satG325R*) induced Sb resistance in both *mrpA−/-* and wild-type parasites, increasing Sb tolerance by 2–3.2-fold	Douanne et al. (2020)
Paromomycin	Visceral Leishmaniasis (VL)	*L. donovani* (Field isolates)	Paromomycin-resistant *L. donovani*	PMM resistance induced at the promastigote level was evident in amastigotes, with a 6-fold decrease in PMM susceptibility. Comparative transcriptome analysis revealed modulated expression of 500 genes in PMM-R parasites	Mechanisms include reduced oxidative phosphorylation, increased glycosomal succinate fermentation, altered energy generation via lipids and amino acids, reduced DNA synthesis, increased DNA repair, and altered protein synthesis/degradation. PMM-R parasites showed increased susceptibility in the presence of Ca2+ channel antagonists, suggesting the involvement of ABC transporters	Verma et al. (2017)

## 5 Genomic plasticity and drug resistance in *Leishmania*

### 5.1 Atypical genome of *Leishmania*

The Leishmania genome exhibits unique characteristics compared to other eukaryotes, with variations in chromosome numbers and gene sets (Iv. Gerasimov et al., 2023). Recent genomic assemblies, such as that of Leishmania major, revealed a 32.8 Mb genome containing 11,238 genes distributed across 36 chromosomes (Camacho et al., 2021). Initially thought to be strictly diploid, *Leishmania* populations display mosaic aneuploidy, where chromosomal copy numbers vary between strains and species (Bussotti et al., 2018; Zackay et al., 2018).

Unlike typical eukaryotes, *Leishmania* genes lack introns and are organized into unidirectional polycistronic transcription units without functional clustering (Bartholomeu et al., 2021). Transcription is constitutive, mediated by RNA polymerase II, but lacks canonical promoters (Saha, 2020). Epigenetic mechanisms, including histone modifications and DNA accessibility, regulate transcription initiation (Chandra et al., 2017), while termination is determined by base J (Reynolds et al., 2016). Since transcriptional regulation is minimal, gene expression is primarily controlled post-transcriptionally via mRNA stability, translation efficiency, and protein degradation (Grünebast and Clos, 2020).

### 5.2 Genetic diversity and genomic plasticity

Large-scale genomic studies have revealed extensive genetic diversity in Leishmania, influencing its geographical distribution and clinical manifestations (Llanes et al., 2022; Franssen et al., 2020). Single-cell sequencing has identified multiple karyotypes within a single clone (Imamura et al., 2020; Negreira et al., 2022), and mixed-genotype infections are common even within the same host tissue (Cupolillo et al., 2020).

#### 5.2.1 Mechanisms of genomic plasticity

**FIGURE 4:**
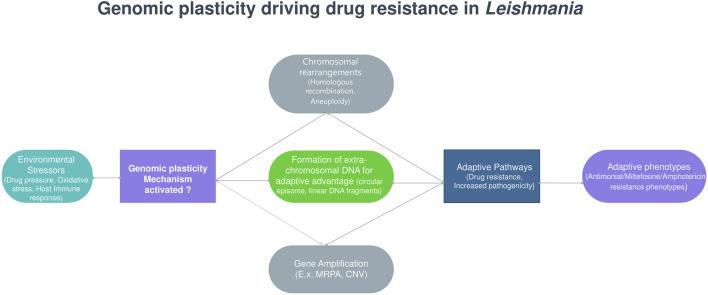
Genomic plasticity driving drug resistance in *Leishmania*.

Large-scale genomic studies have highlighted the extensive genetic diversity in *Leishmania*, which influences its geographical distribution and clinical manifestations (Ruang-Areerate et al., 2023; Hadermann et al., 2023). Single-cell sequencing has revealed multiple karyotypes within a single clone (Imamura et al., 2020; Negreira et al., 2023), and mixed-genotype infections are frequently observed even within the same host tissue (Bharati, 2022). Genomic plasticity in *Leishmania* is driven by several mechanisms, including mosaic aneuploidy—a common feature that enables rapid adaptation under stress, with non-random, strain-specific patterns indicating selective pressure (Sterkers et al., 2014; Dujardin et al., 2014; Bussotti et al., 2018). Additionally, gene copy number variations (CNVs) such as tandem amplifications, deletions, and extrachromosomal circular or linear DNA contribute to genomic diversity (Black et al., 2023). Homologous recombination, facilitated by repeated sequences near DNA double-strand breaks, further promotes gene rearrangements (da Silva, 2021), while telomeric instability due to replicative stress in subtelomeric regions enhances genomic variability (Damasceno et al., 2016). Despite predominantly clonal expansion, evidence suggests genetic exchange between parasites, possibly through sexual recombination, which may enhance long-term survival (Figure 4) (Van den Broeck et al., 2020).

### 5.3 Genomic adaptations and drug resistance

*Leishmania* parasites exhibit remarkable genomic plasticity that underpins their adaptability to drug pressure. The parasite genome demonstrates significant instability characterized by aneuploidy, copy number variations (CNVs), and single nucleotide polymorphisms that collectively facilitate rapid adaptation to therapeutic interventions (Laffitte et al., 2016). These genomic alterations enable *Leishmania* to develop resistance through various mechanisms including altered drug transport, target modification, and enhanced metabolic detoxification pathways (Ponte-Sucre et al., 2017). The genomic instability of *Leishmania* serves as an evolutionary advantage, allowing rapid selection of resistant populations under drug pressure. Whole genome sequencing studies have revealed extensive chromosomal amplifications and deletions occurring in response to drug exposure showing more frequent copy number alterations (Leprohon et al., 2014). These changes often correlate with altered expression of genes involved in stress response, metabolism, and drug transport, establishing a genetic foundation for resistance development (Ubeda et al., 2008).

### 5.4 Gaps in current knowledge and future directions

While genomic plasticity contributes to resistance, the precise evolutionary trajectory of resistant Leishmania strains under drug pressure remains unclear, necessitating longitudinal studies to track genomic changes in response to treatment (Santi and Murta, 2022). Additionally, the role of host immune modulation in drug resistance requires further exploration, as studies suggest that resistant parasites alter host immune responses, though the molecular mechanisms remain poorly characterized (Costa-da-Silva et al., 2022). Beyond genetic mutations, epigenetic modifications such as histone modifications, DNA methylation, and non-coding RNAs may regulate resistance-related genes, warranting further investigation (Afrin et al., 2019). Furthermore, cross-resistance to different drug classes, including miltefosine and amphotericin B, has been observed, yet the underlying mechanisms remain inadequately understood (Zhang et al., 2025). Lastly, while genomic and transcriptomic analyses have identified numerous resistance-associated genes, their functional roles remain speculative, highlighting the need for gene knockout or overexpression studies to validate their contributions (Bharadava et al., 2024).

## 6 Drug pipeline advances in leishmaniasis

Significant global efforts are driving advancements in the leishmaniasis drug pipeline, with clinical trials aiming to refine treatments, enhance prevention strategies, strengthen immune responses, and improve diagnostics These efforts span various forms of the leishmaniasis addressing challenges such as drug resistance, treatment adherence, and accessibility. Recent studies have also emphasized the role of host-directed therapies and novel drug delivery systems to enhance treatment efficacy. In visceral leishmaniasis (VL), treatment trials have emphasized refining the use of Amphotericin B, a highly effective but costly drug. Liposomal Amphotericin B has been tested in single- and multiple-dose regimens, showing strong efficacy, especially in Indian patients; however, its high cost limits its accessibility (Lee et al., 2024). To improve outcomes, combination therapies including Amphotericin B, Miltefosine, and Paromomycin are being evaluated, with trials like NCT01122771 exploring shorter regimens to increase treatment adherence and reduce toxicity (Rahman et al., 2017). For patients co-infected with HIV, Miltefosine has demonstrated potential in Ethiopian trials, though the rise of drug resistance in endemic regions remains a concern (Ritmeijer et al., 2006).

Beyond treatment strategies, significant prevention efforts have been focused heavily on vector control, with long-lasting insecticide-treated nets (LLINs) proving effective in reducing transmission rates in endemic areas (Garlapati et al., 2021). Additionally, trials are assessing immune response modulation with agents like N-Acetylcysteine, often paired with Sodium Stibogluconate, to boost host resistance to infection, offering promise for high-risk individuals (Magalhães et al., 2022). In diagnostics, rapid diagnostic tests (RDTs) and cost-effective assays such as LAMP are being developed to improve early detection and timely treatment, crucial for reducing disease severity and transmission (Erber et al., 2022). New treatment options, like Sitamaquine and lipid-based Amphotericin B formulations, are being tested for patients resistant to traditional drugs (Sundar et al., 2011b). For relapse prevention in HIV co-infected patients, combination therapies with drugs like Pentamidine are also under study (Diro et al., 2015). Vaccination is an emerging focus, with trials for the LEISH-F3 + SLA-SE vaccine indicating favorable safety and immunogenicity in healthy adults, marking an important step toward an immune-based prophylactic for VL (Coler et al., 2015; Lacey et al., 2022). While these advances mark significant progress, several challenges persist, including resistance, high relapse rates, and limited diagnostic resources. Ongoing trials assessing predictive biomarkers and less invasive monitoring methods seek to address these limitations and enhance patient outcomes, though high costs and limited access continue to restrict broader application of effective treatments like liposomal formulations.

While visceral leishmaniasis has been the focus of extensive clinical trials, parallel efforts are advancing treatment strategies for cutaneous leishmaniasis (CL), where pentavalent antimonials remain a frontline choice despite concerns around toxicity and variable efficacy across different regions. These drugs show limited efficacy against certain species, like *L. major* and *Leishmania tropica*, prompting dose optimization trials and alternative delivery methods to reduce side effects. Miltefosine remains a key option, particularly in areas with limited injectable access, though issues of teratogenicity and resistance drive interest in more accessible oral formulations (van Henten et al., 2021). Combination therapies, such as Miltefosine with Paromomycin or liposomal Amphotericin B, are also being investigated to improve effectiveness and reduce treatment duration (Intakhan et al., 2024). Fexinidazole has shown promise as a short-course oral therapy for various forms of leishmaniasis (de Morais-Teixeira et al., 2019).

Nanoparticle formulations of Amphotericin B, including topical options, are being trialed for safer, localized treatment of CL lesions (Fairuz et al., 2022). Adjunct therapies with azole antifungals like Itraconazole are also under evaluation for their immunomodulatory effects, potentially benefiting resistant cases when combined with first-line treatments (Fischer et al., 2024). Immunotherapy is an expanding field, with trials assessing cytokine modulators such as GM-CSF and interferon-gamma alongside conventional treatments to enhance immune response and healing, particularly in immunocompromised patients (Akbari et al., 2021). Advances in diagnostics for CL, focusing on point-of-care tests and molecular tools like qPCR and LAMP, support accurate, early detection, enabling species-specific treatment in resource-limited settings (Erber et al., 2022).

## 7 Emerging drug targets in *Leishmania*

Recent advances in molecular and cellular biology have identified several promising drug targets that could pave the way for novel therapeutic interventions (Jain et al., 2022). One notable target is cyclin-dependent kinase 12 (CDK12), whose inhibition has demonstrated efficacy against *Leishmania* parasites, suggesting its potential as a therapeutic target for visceral leishmaniasis (Wyllie et al., 2018). Another promising target is the cytochrome bc1 complex, with inhibitors disrupting mitochondrial function in *Leishmania* species, leading to parasite death (Saldivia et al., 2024). Additionally, the proteasome has been identified as a viable target, with inhibitors like GNF6702 exhibiting broad-spectrum antiprotozoal activity against *Leishmania* species by selectively targeting the parasite’s proteasome without affecting host cells (Khare et al., 2016). Enzymes in the purine salvage pathway, such as adenine phosphoribosyltransferase (APRT) and hypoxanthine-guanine phosphoribosyltransferase (HGPRT), are essential for *Leishmania* survival and have been explored as potential targets (Boitz et al., 2012). Another critical pathway is the trypanothione system, which is unique to trypanosomatids and replaces the glutathione system in these parasites. Inhibitors of trypanothione reductase (TR) and trypanothione synthetase (TryS) have shown potent antileishmanial activity in preclinical studies (Beniwal et al., 2025; González-Montero et al., 2024). Additionally, sterol biosynthesis in *Leishmania* has emerged as a promising target. The enzyme sterol 14α-demethylase (CYP51), which is involved in ergosterol biosynthesis, has been successfully targeted by azole compounds, such as posaconazole and ketoconazole (Bhusal et al., 2024; Emami et al., 2017). Furthermore, protein kinases, particularly mitogen-activated protein kinases (MAPKs) and cyclin-dependent kinases (CDKs), play crucial roles in *Leishmania* proliferation and differentiation, making them attractive targets for kinase inhibitors (Naula et al., 2005). Other emerging targets include leishmanial proteases, such as cysteine proteases (e.g., CPA, CPB) and metalloproteases, which are involved in parasite virulence and immune evasion (de Oliveira et al., 2025). Polyamine biosynthesis in Leishmania represents another promising drug target, as enzymes like ornithine decarboxylase (ODC) and spermidine synthase are critical for parasite survival. Inhibiting key steps in polyamine metabolism could disrupt growth and redox balance in the parasite, offering potential therapeutic strategies for leishmaniasis (Carter et al., 2022). Finally, host-directed therapies that modulate immune responses, such as targeting host cytokines (e.g., IL-10, TGF-β) or enhancing macrophage leishmanicidal activity, represent a promising complementary approach (Kumar et al., 2017). Despite these advancements, significant research gaps remain. These include a lack of understanding of drug resistance mechanisms, the need for better *in vitro* and *in vivo* models, and the absence of effective vaccines. Addressing these gaps will require interdisciplinary collaboration, increased funding, and the integration of omics technologies to identify novel biomarkers and therapeutic targets.

## 8 Conclusion

The emergence of multi-drug resistant *Leishmania* strains presents a major challenge to disease management, significantly compromising clinical outcomes. The parasite’s unique genomic dynamics—manifested through mechanisms like gene amplifications, chromosomal rearrangements, and mosaic aneuploidy—facilitates rapid adaptation to pharmacological stress and drives phenotypic diversity. This genomic flexibility, coupled with specific mutations in drug targets, overexpression of efflux transporters, and alterations in sterol biosynthesis pathways, enables *Leishmania* to withstand various therapeutic interventions and develop resistance to multiple drugs.

Recent advances in understanding the molecular underpinnings of resistance highlight the critical role of genomic plasticity in the rapid emergence of resistant phenotypes. This knowledge underscores the urgent need for innovative therapeutic strategies that go beyond traditional approaches. By integrating genomic insights with advanced drug discovery techniques, it is possible to design targeted interventions, such as combination therapies and inhibitors that specifically disrupt resistance pathways or exploit the parasite’s genomic vulnerabilities.

Despite significant progress, the effective translation of these findings into clinical practice remains a challenge. *Leishmania*’s inherent genomic complexity and adaptability necessitate a multifaceted approach that combines genetic, biochemical, and pharmacological perspectives. Future research should aim to deepen our understanding of drug resistance mechanisms, with a focus on developing precision medicines tailored to specific resistance profiles. Additionally, exploring combination therapies or repurposing existing drugs to counteract known resistance mechanisms may offer promising avenues for managing resistant *Leishmani*a cases. Enhanced diagnostic tools for early detection of resistant strains, coupled with therapeutic strategies that disrupt key resistance pathways, will be essential to ensuring the long-term success of anti-leishmanial therapies and improving patient outcomes. Expanding our understanding of molecular adaptations across different *Leishmania* species and strains will be key to designing next-generation therapies and sustainable treatment regimens.

## Abbreviations

VL, Visceral Leishmaniasis; CL, Cutaneous Leishmaniasis; SSG, Sodium Stibogluconate; AmB, Amphotericin B; L-AmB, Liposomal Amphotericin B; MIL, Miltefosine; PM, Paromomycin; MRPA, Multidrug Resistance Protein A; AQP1, Aquaglyceroporin 1; PKDL, Post-Kala-Azar Dermal Leishmaniasis; HSP, Heat Shock Protein; ABC, ATP-Binding Cassette; MDR1, Multidrug Resistance 1; MSL, Miltefosine Sensitivity Locus; SMT, Sterol Methyltransferase; SC5D, Sterol C5-Desaturase; RDT, Rapid Diagnostic Test; LAMP, Loop-Mediated Isothermal Amplification; CNV, Copy Number Variation; SNP, Single Nucleotide Polymorphism; LLINs, Long-Lasting Insecticide-Treated Nets.
